# Plasmocytome solitaire osseux: expérience de l'institut national d'oncologie de Rabat (INO)

**DOI:** 10.11604/pamj.2014.17.180.2604

**Published:** 2014-03-07

**Authors:** Adama Diakité, Karima Nouni, Sara Bellefqih, Tayeb Kebdani, Noureddine Benjaafar

**Affiliations:** 1 Service de Radiothérapie, Institut National d'Oncologie Sidi Mohamed Ben Abdellah de Rabat-Sale, Rabat, Maroc

**Keywords:** Plasmocytome solitaire osseux, radiothérapie, myelome multiple, Solitary plasmacytoma of bone, radiotherapy, multiple myeloma

## Abstract

Nous évaluerons les aspects diagnostiques, thérapeutiques et évolutifs du plasmocytome solitaire osseux dans notre structure. Il s'agit d'une revue rétrospective portant sur 11 patients suivis et traités dans le Service de Radiothérapie de l'Institut National d'Oncologie de Rabat pour un plasmocytome solitaire osseux entre janvier 1999 et décembre 2009. L’âge moyen de nos patients était de 53 ans (19-79 ans). La douleur était le signe révélateur le plus fréquent. Le siège des lésions était rachidien dans 4cas, nasosinusien dans 2cas, orbitaire dans 1 cas, costal dans 1 cas, presternal dans 1 cas, sellaire dans 1 cas et sacré dans 1 cas. Tous les malades ont reçu une radiothérapie. Cette irradiation était délivrée seule dans 8 cas (73%) ou associée à la chirurgie (laminectomie) dans 3 cas (27%). La dose moyenne de la radiothérapie était de 44 Gy (30 à 60 Gy) et celle-ci a été délivrée par un appareil de télécobalthérapie ou un accélérateur linéaire. La durée moyenne de suivi était de 78 mois (38-118 mois). Le contrôle local, défini par une stabilité radiologique, a été obtenu chez 7 des 11 malades (87%). Un patient a évolué vers un myélome multiple après un délai de 8 mois. La radiothérapie constitue un traitement efficace du plasmocytome osseux solitaire. Le pronostic est affecté par l’évolution vers le myélome multiple, ce qui justifie une surveillance rigoureuse après traitement et suggère une réflexion sur la place exacte de la chimiothérapie.

## Introduction

Le plasmocytome solitaire est une tumeur plasmocytaire rare [1]. Il regroupe deux entités distinctes en rapport avec la localisation tumorale: soit l'os (plasmocytome solitaire osseux) ou le tissu mou (plasmocytome solitaire extra médullaire) [2]. Le plasmocytome osseux solitaire se caractérise par une lésion unique affectant le plus souvent le squelette axial notamment les vertèbres. Son diagnostic repose sur la confirmation histologique de la prolifération plasmocytaire, l′absence de sa diffusion médullaire et le caractère unique de la lésion. L’évolution fréquente du plasmocytome solitaire osseux vers le myélome multiple, décrite dans la littérature dans 55%, des cas remet toujours en question son authenticité nosologique [3]. Le traitement de référence est la radiothérapie, parfois associée à la chirurgie. La place de la chimiothérapie reste à définir.

## Méthodes

Il s'agit d'une revue rétrospective portant sur 11 patients suivis et traités dans le Service de Radiothérapie de l'Institut National d'Oncologie de Rabat pour un plasmocytome solitaire osseux entre janvier 1999 et décembre 2009. Ont été inclus dans cette étude tous les patients qui présentaient les critères suivants: Une biopsie de la lésion osseuse positive, permettant de poser le diagnostic de tumeur plasmocytaire; un bilan radiologique permettant d'affirmer le caractère unique de la lésion osseuse; une plasmocytose médullaire inférieure à 10%. Le bilan initial comprenait en outre une électrophorèse des protides, une immunoélectrophorèse des protides, une numération formule sanguine, et la recherche d'une protéinurie de Bence- Jones. Ont été exclus les patients ne présentant pas l'ensemble de ces critères nécessaires pour le diagnostic de certitude.

## Résultats

Après analyse des résultats de notre étude, l’âge moyen de nos patients était de 53 ans (19-79 ans). Il s'agissait de 6 femmes et 5 hommes, le sexe ratio était 1,5 F/H. Le délai moyen de diagnostic était de 8 mois (3-24 mois). La douleur était le signe révélateur le plus fréquent (10 cas) suivi de la compression médullaire (3cas), la diplopie avec Hypertension Artérielle (3cas), l'obstruction nasale(2cas) et l'exophtalmie(1 cas). Le siège des lésions était rachidien dans 4cas, nasosinusien dans 2cas, orbitaire dans 1 cas, costal dans 1 cas, presternal dans 1 cas, sellaire dans 1 cas et sacré dans 1 cas.

Tous les malades ont reçu une radiothérapie. Cette irradiation était délivrée seule dans 8 cas (73%) ou associée à la chirurgie (laminectomie) dans 3 cas (27%). La dose moyenne de la radiothérapie était de 44 Gy (30 à 60 Gy) et celle-ci a été délivrée par un appareil de télécobalthérapie ou un accélérateur linéaire. Le volume cible était la lésion osseuse avec une marge de sécurité (une ou deux vertèbres de part et d'autre en cas d'atteinte rachidienne et 4 à 5 cm de la lésion radiologique dans les autres cas).

La durée moyenne de suivi était de 78 mois (38-118 mois). Le suivi était biologique (hémogramme, immuno-électrophorèse des protides, vitesse de sédimentation) et radiologique.

Le contrôle local, défini par une stabilité radiologique, a été obtenu chez 7 des 11 malades (87%). La rechute locale caractérisée par la reprise évolutive biologique et la modification radiologique sur les contrôles successifs n'a été rétrouvé chez aucun de nos patients. De même, la multifocalité définie par l'apparition d'une ou plusieurs lésions sans plasmocytose médullaire n'a pas été observé dans notre population.

La transformation myélomateuse a été retenue si, en plus d'autres lésions osseuses, la ponction sternale ou la biopsie médullaire montrait une plasmocytose de plus de 10%. Un patient a évolué vers un myélome multiple après un délai de 8 mois. Trois patients ont été perdus de vue après la fin de traitement.

## Discussion

Le plasmocytome solitaire osseux (POS) est défini comme une lésion focale composée de plasmocytes malins mais sans envahissement médullaire diffus, ce qui le distingue du myélome multiple. Il touche principalement le squelette axial.

Un certain nombre de critères sont nécessaires pour porter le diagnostic de plasmocytome solitaire: l'absence d'anémie, un bilan phosphocalcique normal, une fonction rénale normale, une cytologie médullaire normale dans au moins deux sites différents et une absence d'autres lésions sur les radiographies du squelette ou sur l'IRM.

Le traitement du PSO repose essentiellement sur la radiothérapie (RT); il s'agit de tumeurs radiosensibles et radio curables [4, 5]. Dans notre série, tous les patients ont été traités par la radiothérapie avec un bon contrôle local (87%).

Mais la question de la dose optimale de radiothérapie reste posée puisque la littérature ne tranche pas sur la relation dose-effet de façon claire. Mendenhal et al [6] ont suggéré une dose minimale de 40 Gy pour obtenir un bon contrôle local. Ils ont rapporté un taux de contrôle local de 94% à des doses supérieures à 40 Gy contre 69%si les doses étaient plus faibles. Frassica et al [7], dans une série de 46 malades, ont rapporté un taux d’échec local de 15,6% si la dose de radiothérapie était inférieure à 45 Gy, alors que le taux de contrôle local était de 100% si la dose était supérieure. Certains auteurs proposent une dose de 40-50 Gy pour les petites tumeurs et des doses plus élevées pour les tumeurs de grande taille [8, 9].

Ainsi Tsang et al [10] ne retrouvent aucun effet dose au-delà de 35 Gy pour les petites tumeurs et suggèrent de réserver les fortes doses ou un traitement combiné pour les tumeurs bulky.

Dans notre série avec une dose moyenne de 44 Gy nous avons noté un bon contrôle local; mais notre effectif faible ne permet pas de tirer une conclusion sur la relation dose-effet.

La place de la chirurgie dans le PSO est très limitée. Réalisée souvent à visée diagnostique, elle ne doit pas être étendue et mutilante. Elle ne se justi'e qu'en cas de laminectomie à visée décompressive dans les localisations rachidiennes sans preuve histologique préalable ou pour une réparation orthopédique en cas de fracture ou menace de fracture, permettant ainsi de réaliser la radiothérapie dans des conditions confortables [3]. Dans notre série trois patients ont eu une chirurgie à type de laminectomie.

La place de la chimiothérapie reste controversée. Une seule étude prospective a rapporté un bénéfice avec l'association chimiothérapie et la RT par rapport à la RT seule [11]. La chimiothérapie utilisée était du melphalan-prednisone administrée toutes les six semaines pendant trois ans. Même si un nombre limité de patients a été inclus (n = 53), la radio chimiothérapie combinée semblait dans cette étude améliorer la rémission et la durée de survie. D'autres études ne retrouvent pas son utilité, et certains auteurs considèrent qu'elle ne permet pas d’éviter la survenue du myélome multiple et qu'au maximum elle pourrait en retarder le délai d'apparition [12]. D'autres concluent même à son inefficacité [13, 14]. Aucun de nos patients n'a reçu de chimiothérapie.

Les facteurs prédictifs de la récidive locale semblent être: les doses insuffisantes de radiothérapie, le siège du plasmocytome solitaire osseux, les lésions rachidiennes ou pelviennes étant plus difficiles à traiter que les lésions périphériques, et la taille tumorale [3].

L’évolution vers la multifocalité des lésions est une situation rare et discutée, elle a été décrite dans 2 à 15% des cas dans la littérature [4, 7, 13, 14]. Il s'agit de nouvelles localisations osseuses à distance de la première et ne correspondant pas à une dissémination myélomateuse, mais à un plasmocytome multicentrique. Toutefois, la distinction avec un myélome multiple n'est pas toujours aisée, surtout qu'une simple ponction de moelle n'exclut pas formellement une dissémination [3, 15].

Le pronostic du plasmocytome solitaire osseux reste dominé par le risque de survenue d'un myélome multiple. Dans la littérature, la progression vers le myélome multiple se produit habituellement dans les deux ans suivant le diagnostic initial, mais il a été noté des cas jusqu′à 15 ans plus tard indiquant la nécessité du suivi à long terme des patients [16–21]. Dans notre série un seul patient a évolué vers un myélome multiple après 8 mois.

Après traitement du PSO, la surveillance doit donc être minutieuse et durable. L'examen clinique, la radiographie, la VS, l'immunoélectrophorèse des protides, et le médullogramme sont les éléments de surveillance.

## Conclusion

La radiothérapie constitue un traitement efficace du plasmocytome osseux solitaire. Elle permet un contrôle local dans 90% des cas. Le pronostic est affecté par l’évolution vers le myélome multiple, ce qui justifie une surveillance rigoureuse après traitement et suggère une réflexion sur la place exacte de la chimiothérapie.
